# Prevention of Suicidal Behavior in Prisons: An Overview of Initiatives Based on a Systematic Review of Research on Near-Lethal Suicide Attempts

**DOI:** 10.1027/0227-5910/a000394

**Published:** 2016-06-09

**Authors:** Lisa Marzano, Keith Hawton, Adrienne Rivlin, E. Naomi Smith, Mary Piper, Seena Fazel

**Affiliations:** ^1^ Department of Psychology, Middlesex University, London, UK; ^2^ Centre for Suicide Research, University of Oxford, UK; ^3^ Health and Justice, Health and Wellbeing Directorate, Public Health England, London, UK

**Keywords:** suicide, prison, prevention, jail, self-harm

## Abstract

**Abstract.** *Background:* Worldwide, prisoners are at high risk of suicide. Research on near-lethal suicide attempts can provide important insights into risk and protective factors, and inform suicide prevention initiatives in prison. *Aims:* To synthesize findings of research on near-lethal attempts in prisons, and consider their implications for suicide prevention policies and practice, in the context of other research in custody and other settings. *Method:* We searched two bibliographic indexes for studies in any language on near-lethal and severe self-harm in prisoners, supplemented by targeted searches over the period 2000–2014. We extracted information on risk factors descriptively. Data were not meta-analyzed owing to heterogeneity of samples and methods. *Results:* We identified eight studies reporting associations between prisoner near-lethal attempts and specific factors. The latter included historical, prison-related, and clinical factors, including psychiatric morbidity and comorbidity, trauma, social isolation, and bullying. These factors were also identified as important in prisoners' own accounts of what may have contributed to their attempts (presented in four studies). *Conclusion:* Factors associated with prisoners' severe suicide attempts include a range of potentially modifiable clinical, psychosocial, and environmental factors. We make recommendations to address these factors in order to improve detection, management, and prevention of suicide risk in prisoners.

Reducing the number of suicides in jails and prisons is an international priority (World Health Organization, 2007) and many countries have national standards and guidelines for suicide prevention in custodial settings (Daigle et al., 2007). Suicide remains one of the most common causes of death in custody worldwide, with rates substantially higher than in the general population (Fazel, Grann, Kling, & Hawton, 2011). Studies of trends in prison suicides in Germany (Opitz-Welke, Bennefeld-Kersten, Konrad, & Welke, 2013), Italy (Cinosi, Martinotti, De Risio, & Giannantonio, 2013), and other countries in the European Union (Rabe, 2012) as well as Australia (Kariminia et al., 2007) and the US (Baillargeon et al., 2009) suggest that current suicide prevention strategies need improving in order to better meet the complex needs of the prison population. 

Several prison suicide prevention strategies, including those in the US, UK, and Australia, have been developed partly in response to what is known about the epidemiology of suicide in prisoners and in-depth analyses of the prison and clinical records of inmates thought to have taken their own lives (Konrad et al., 2007). These strategies need updating as new findings about suicide in prisoners emerge. Research conducted with prisoners who have made near-lethal suicide attempts – in other words, medically severe and potentially deadly attempts (Magaletta, Patry, Wheat, & Bates, 2008) – can substantially enrich our knowledge of what is likely to be effective in preventing suicidal behavior in prisons (Marzano, Rivlin, Fazel, & Hawton, 2009). As well as representing an important problem in their own right, near-lethal suicide attempts have been shown to provide a valid proxy for completed suicide in prisoners (Rivlin, Fazel, Marzano, & Hawton, 2012).

Interviewing those who have engaged in near-lethal suicide attempts can provide insights into risk factors and the suicidal process, which is not possible through analyses of official records or interviews with staff or informants. Such an approach is likely to contribute to a richer understanding of the ways in which contributory and protective factors interact, and their relative importance in the pathways leading to suicidal behavior. In turn, this information may help identify and prioritize evidence-based preventative initiatives. 

Therefore, we conducted a systematic review of the literature on near-lethal suicide attempts in prisoners. We provide an overview of this research and discuss its implications for suicide prevention policies and practices in the context of other relevant literature on suicide in other offending groups (including those in police custody and recently released prisoners).

## Method 

### Search Strategy and Inclusion Criteria

We searched titles and abstracts of MEDLINE and PsycINFO from January 1, 2000 to January 1, 2014 using the following terms: Near-lethal self-harm OR Near-fatal self-harm OR Suicide OR Suicid* OR Suicide Attempt OR Severe Self-mutilation OR Severe Deliberate self-harm" AND "Prison* OR Custody OR Jail OR Police*. Further targeted searches, including hand-searches of relevant reference/citation lists, were undertaken with Google Scholar. We included articles relevant to near-lethal suicide attempts in prisoners, both published and unpublished, with no language restrictions. We extracted information on risk and contributing factors, methods, and lethality of attempts. Where applicable, we also extracted information on potential preventive factors, based on the accounts of prisoners involved in near-lethal attempts. We excluded studies focused on completed suicide alone (which are reviewed elsewhere; Fazel, Cartwright, Norman-Nott, & Hawton, 2008), studies that did not provide information on the severity or lethality of suicide attempts and those focused on suicidal ideation alone. Studies conducted in any setting other than prisons were excluded. Eligible studies were screened independently by two authors (E. N. S. and S. F.). There were no disagreements between the authors when screening eligible articles. 

In the Results section we present the findings from the studies reviewed, and then consider their implications for suicide prevention in the Discussion.

## Results 

### The Included Studies

**Figure 1:**
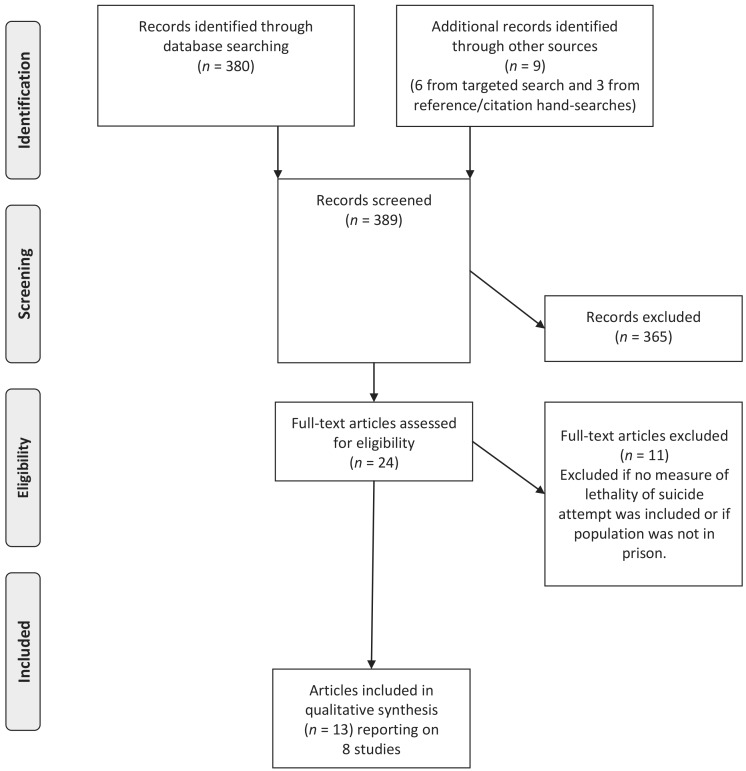
PRISMA flow diagram. Results of search for articles focusing on near-lethal suicide attempts in prisoners. Adapted from Moher et al., 2009.

Out of 389 articles identified in our search, 13 papers met our inclusion criteria, based on eight separate studies published between 2000 and 2014 (see Table 1 and PRISMA flowchart, Figure 1). Three studies were conducted in the US (Bonner, 2006; Magaletta et al., 2008; Suto & Arnaut, 2010), three in England and Wales (Borrill, Snow, Medlicott, Teers, & Paton, 2005; Marzano, Fazel, Rivlin, & Hawton, 2010; Marzano, Fazel, Rivlin, & Hawton, 2011; Marzano, Hawton, Rivlin, & Fazel, 2011; Rivlin, Hawton, Marzano, & Fazel, 2010, 2013; Rivlin, Fazel, Marzano, & Hawton, 2011; Rivlin, Ferris, Marzano, Fazel, & Hawton 2013), one in The Netherlands (Blaauw, Kerkhof, & Winkel, 2001) and one in Germany (Lohner & Konrad, 2006). Prisoner compositions varied between studies (e.g., two studies included only female prisoners; two included mixed but predominantly male samples; and the remaining four focused only on male prisoners), as did samples sizes (ranging from 15 to 274) and outcomes. For example, three studies were mostly focused on self-harm incidents involving different levels of lethality (including comparisons between high- and low-lethality self-harm) in relation to a restricted range of variables (Bonner, 2006; Lohner & Konrad, 2006; Magaletta et al., 2008). By contrast, our own studies of near-lethal self-harm (Marzano et al., 2010; Marzano, Fazel, et al., 2011; Marzano, Hawton, et al., 2011; Rivlin et al., 2010, 2011; Rivlin, Ferris, et al., 2013; Rivlin, Hawton, et al., 2013) and the work of Blaauw in The Netherlands (Blaauw et al., 2001) investigated differences between prisoners who had made serious suicide attempts and prisoners who had not (but with slightly different operational definitions). The remaining two studies were qualitative studies of prisoners who had attempted suicide in prison, with no comparison groups (Borrill et al., 2005; Suto & Arnaut, 2010). 

**Table 1:** Research on near-lethal attempts in prisoners

Article	Country	Sample	Reported association(s) with near-lethal attempts
Blaauw, E., Winkel, F. W., & Kerkhof, A. J. (2001)	The Netherlands	274 prisoners (92% male), of which 53 suicidal prisoners and 221 nonsuicidal controls.	Bullying (especially serious bullying) more prevalent among inmates with a serious suicide attempt. The latter group, compared with controls, included more inmates charged with sex offences; with a history of psychiatric treatment and held in a special unit for mentally disordered or vulnerable prisoners (but no difference in proportion of young inmates; prisoners charged with a violent offence; in jail for more than 6 weeks; with a history of drug abuse; and previous incarceration).
Bonner, R. L. (2006)	US	134 male inmates from a medium-security federal prison.	Significant interactions between anticipated segregation stress and hopelessness, mental health problem history, and suicide attempt lethality history.
Borrill, J., Snow, L., Medlicott, D., Teers, R., & Paton, J. (2005)	England and Wales	15 women (adults and young offenders) – no control group.	Identified vulnerability and precipitating factors included: trauma and loss, mental health problems, children and family issues, drug use, detoxification, and bullying.
Lohner, J., & Konrad, N. (2006)	Germany	49 male prisoners (remand and sentenced prisoners; adults' detention and young offender institution). Of these, 16 were classified as having made a "serious attempt" (with regard to intent and lethality).	Significant positive association between depression and hopelessness and suicidal intent and lethality.
Magaletta, P. R., Patry, M. W., Wheat, B., & Bates, J. (2008)	US	205 male inmates in federal custody.	Increases in suicide attempt lethality associated with the presence of Axis II disorders, favorable staff interactions, and decreased use of drugs other than marijuana, alcohol, cocaine, or depressants.
Marzano et al. (2010, 2011a, 2011b)	England and Wales	60 female prisoners and 60 control prisoners (with no history of near-lethal attempts in custody), matched by age and gender.	Near-lethal self-harm associated with the following factors:*Sociodemographic:* no educational qualifications. *Criminological:* prior prison spell (vs. none); remand status; less than 30 days in current establishment; in single cell; in a safer cell; not on normal wing location; imprisonment perceived as difficult. *Psychiatric and medical:* current major depression, panic disorder, social anxiety, PTSD, obsessive-compulsive disorder, psychotic disorder, comorbidity; past major depression, psychotic disorder, comorbidity; serious physical illness; history of in-patient and out-patient treatment; previous self-harm (in prison and outside). *Psychological:* hopelessness; low self-esteem; impulsivity; aggression; hostility. *Adverse life events:* childhood trauma (incl. emotional abuse and neglect; physical abuse and neglect; sexual abuse); family history of suicide; death of partner or child; local authority care under the age of 16; run away from home; violence in the home; money problems; adverse life event in last six months. *Social support:* poor social support; no close or good friends outside prison.
Suto, I., & Arnaut, G. L. (2010)	US	24 inmates in state prison facilities (of which three females) – no control group.	Identified precipitating factors included: mental health issues, relationship problems, and prison factors.
Rivlin et al. (2010, 2011, 2013a, 2013b)	England and Wales	60 male prisoners and 60 control prisoners (with no history of near-lethal attempts in custody), matched by age, gender and establishment type.	Near-lethal self-harm associated with the following factors:*Sociodemographic:* White; no educational qualifications. *Criminological:* prior prison spell (vs. none); two or more prior prison sentences; young at first conviction; less than 30 days since first reception and in current establishment; held in a safer cell; unemployed whilst in prison; imprisonment perceived as difficult. *Psychiatric and medical:* current major depression, panic disorder, social anxiety; PTSD, drug misuse, psychotic disorder, comorbidity; past major depression, psychotic disorder, comorbidity; serious physical illness; history of in-patient and out-patient psychiatric treatment; previous self-harm (in prison and outside). *Psychological:* hopelessness; low self-esteem; impulsivity; aggression; hostility. *Adverse life events:* childhood trauma (incl. emotional abuse and neglect; physical neglect); family history of self-harm and/or suicide; bullying; homelessness; death of a parent or sibling; local authority care under the age of 16; adverse life event in last year. *Social support:* poor social support; no close or good friends outside prison; no close or good friends inside prison.

These differences between the studies limit the extent to which the findings lend themselves to direct comparison. Also, the heterogeneity of the samples and factors studied precluded meta-analysis. Nevertheless, there are some consistent findings. These include the key role of mental health problems (including depression and hopelessness), relationship issues (including with children and family), and prison factors (such as bullying, moves within the prison, and employment- or activity-related difficulties).

### Near-Lethal Suicide Attempts 

In most cases the near-lethal acts appeared to have been carried out with high suicidal intent (Lohner & Konrad, 2006; Marzano et al., 2010; Rivlin et al., 2010), when the prisoner was alone in his/her cell. Four studies provided details of the method used, with hanging and ligaturing as the most prevalent (Borrill et al., 2005; Lohner & Konrad, 2006; Marzano et al., 2010; Rivlin et al., 2010).

### Factors Associated With Near-Lethal Suicide Attempts

Where sociodemographic factors are reported, the majority of prisoners involved in near-lethal suicide attempts were similar to the wider prison population, being aged between 18 and 53, single (Marzano, Hawton, et al., 2011; Rivlin, Hawton, et al., 2013), heterosexual (Suto & Arnaut, 2010), and White (Marzano, Hawton, et al., 2011; Rivlin, Hawton, et al., 2013; Suto & Arnaut, 2010). (In England and Wales, three quarters of prisoners are White; Berman & Dar, 2013.) However, aside from poorer educational qualifications, sociodemographic factors were not clearly associated with near-lethal self-harm. 

A number of independent risk factors for near-lethal suicide attempts have been found in male and female prisoners. These include historical (or lifetime) factors that may make a person vulnerable to suicide (e.g., childhood trauma), prison-related factors, and clinical characteristics. 

### Historical Factors 

A number of studies have found that those making near­lethal attempts in prison are more likely than other prisoners to have a history of prior self-harm and suicide attempts (both in prison and outside), and to have received psychiatric hospital inpatient and outpatient treatment (Blaauw et al., 2001; Bonner, 2006; Marzano et al., 2010; Rivlin et al., 2010). Other historical factors relate to adverse life events (Borrill et al., 2005; Suto & Arnaut, 2010), including a family history of suicide (Marzano, Hawton, et al., 2011; Rivlin, Hawton, et al., 2013). 

### Prison-Related Factors

In relation to prison-related factors, there are some inconsistencies in the literature regarding the potential role of historical factors such as a prisoner's prior conviction – a risk factor in the Oxford studies (Marzano, Hawton, et al., 2011; Rivlin, Hawton, et al., 2013), but not significantly associated with high severity attempts in other research (Blaauw et al., 2001) – or specific index offences (Blaauw et al., 2001; Marzano, Hawton, et al., 2011; Rivlin, Hawton, et al., 2013; Suto & Arnaut, 2010). However, findings about prisoners' current experiences of incarceration are fairly consistent. Typically these appear to be significantly more negative than those of control prisoners (Blaauw et al., 2001; Marzano, Hawton, et al., 2011; Rivlin, Hawton, et al., 2013), despite evidence that the interactions with staff of prisoners involved in high-lethality attempts may be more favorable than those of prisoners engaging in low-severity self-harm (Magaletta et al., 2008). Those making near-lethal attempts were also found to have spent less time in custody and/or in their current prison than control prisoners (Marzano, Hawton, et al., 2011; Rivlin, Hawton, et al., 2013). 

### Clinical and Psychosocial Factors

Mental health problems, both current and historical, were specifically identified as factors associated with, and potentially precipitating, near-lethal suicide attempts in prisoners in all eight studies included. Compared with controls, male cases in the Oxford studies were disproportionately affected by major depressive symptoms (see also Lohner & Konrad, 2006), psychosis, anxiety (including posttraumatic stress disorder [PTSD]) and drug misuse disorders, while female cases were more likely than controls to be suffering from major depression, anxiety disorders (53% met criteria for PTSD), and psychosis. In both men and women, comorbidity of disorders was common and significantly associated with near-lethal attempts (Marzano et al., 2010; Rivlin et al., 2010). There were high levels of self-reported aggression, impulsivity, hostility, childhood trauma, and hopelessness (the latter also being a significant risk factor in other research; Lohner & Konrad, 2006), and lower levels of social support and self-esteem (Marzano, Hawton, et al., 2011; Rivlin, Hawton, et al., 2013). 

### Prisoners' Accounts of Their Own Near-Lethal Attempts and Suggestions for Prevention

Four of the studies we reviewed included prisoners' views of the factors contributing to their attempts. In line with the evidence presented in the previous section, these included prison-related difficulties, past trauma, mental health issues, and relationship problems, particularly relating to feelings of loss and rejection, and bullying:

I'd just been sentenced on the Thursday …and I was due to get shipped out two days after…I hadn't got my head around the fact that my sentence was a lot more than what I thought it would be. (Prisoner quoted in Marzano, Hawton, et al., 2011, p. 877)

I believe it was my girlfriend leaving me... I believe that was the last straw that did it. (Prisoner quoted in Suto & Arnaut, 2010, p. 299)

At that time I were [sic] getting upset because I were [sic] hearing voices what were telling me to hurt myself and at the end of the day I could not say no to them. I can't say no to them 'cause they just get to me more and more and more. (Prisoner quoted in Rivlin et al., 2011, p. 313)

In many cases, it was the co-occurrence of several adverse events and feelings that prisoners said contributed to the near-lethal act:

I'd lost my job. I split up with the missus. I had just been run over and beat up by the police. I was back in jail and I made a promise that I would never come back. Missing my baby and that. Just everything all at once. (Prisoner quoted in Rivlin et al., 2011, p. 311)

In three of the studies, prisoners' views about factors that may have prevented their acts were presented. In the Oxford studies over half the prisoners in the male and female samples reported that their attempts could have been prevented (Marzano, Fazel, et al., 2011; Rivlin et al., 2011). Being able to talk to someone was the most frequently cited suggestion for prevention, both in the context of informal peer and staff support, and as part of a counseling intervention:

[…] Some counseling. Someone to get into my head, try to talk to me, try and get round why I am doing these stupid things, try and help me get myself sorted out, get me back to the person I was 3 years ago. (Prisoner quoted in Rivlin et al., 2011, p. 320)

The importance of talking to someone – and being listened to – was also a major theme among the women prisoners interviewed by Borrill et al. (2005). Further recommendations in the three studies include: improvements to the general prison regime (e.g., more time of out cell, sharing a cell with another prisoner); training and support for staff; specialist help for those affected by trauma and mental illness; improved access to and administration of medication; and better support following stressful life events.

## Discussion 

We conducted a systematic review of recent studies of near-lethal suicide attempts in prisoners. Consistent with a stress-diathesis model of suicidal behavior (Mann, 2003), our review suggests that prisoners' serious suicide attempts were not the result of a single cause or event, but due to the accumulation and interaction of both proximal and distal factors, including individual state- and trait-dependent factors, and environmental influences. This has also been reported in studies of suicide in the general population (Hawton & Van Heeringen, 2009) and in other research in prisoners (Jenkins et al., 2005), including studies of completed suicide (Dooley, 1990; Fazel et al., 2008; Fazel, Wolf, & Geddes, 2013). 

**Figure 2:**
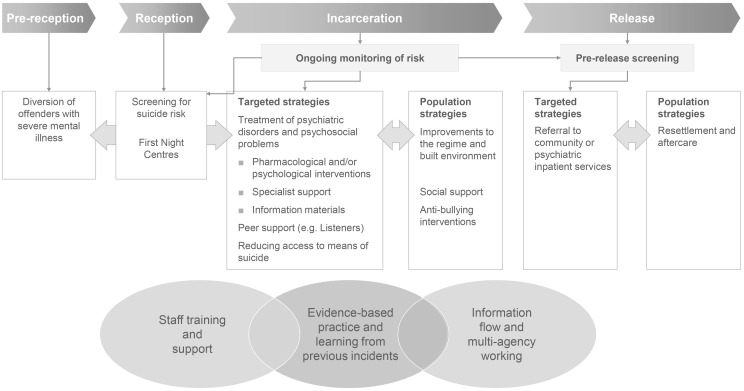
Prevention of suicidal behavior in prisoners.

An important implication of this review is that factors associated with prisoners' suicide attempts include potentially modifiable clinical, psychosocial, and environmental factors. Strategies to reduce self-harm and suicide in prisoners should therefore include attention to these factors, and their interactions. Potential prevention initiatives are presented in Figure 2.

### Improved Detection of Offenders Most at Risk of Suicide

In the Oxford studies of near-lethal attempts, only 24 (40%) male prisoners had made a near-lethal suicide attempt while being on a risk management document (as a result of the current suicide risk assessment process; Rivlin et al., 2010). Similarly, in a recent study in England and Wales almost half (46%) the prisoners who had died by suicide between 2005 and 2008 had never been on an open risk management document during their prison term (Humber, Webb, Piper, Appleby, & Shaw, 2013). This suggests that there are deficiencies in risk assessment and identification, at least in prisons in England and Wales, although the limitations of suicide risk assessment, particularly the high rates of false positives, will need to be considered (National Institute for Clinical Excellence, 2011).

#### Pre-Reception Screening and Diversion of Mentally Disordered Offenders

All the studies reviewed identified strong associations between near-lethal self-harm and mental disorders. This underscores the importance of screening for mental disorder, as well as specifically for suicidality – ideally as early as possible in the criminal justice pathway, to enable diversion from custody of offenders with severe mental illness to alternatives such as secure hospitals, community sentences, or treatment orders. Carrying out a comprehensive triage and assessment process when offenders first come into contact with the police, with support from specialist mental health services, has been recommended for complex psychiatric disorders (Bradley, 2009; see also Kovasznay, Miraglia, Beer, & Way, 2004), although evidence from trials demonstrating whether this is effective is currently lacking.

#### Reception Screening

In the studies we reviewed, near-lethal attempts were associated with high suicide intent, and occurred early on during custody. This supports the findings of previous studies showing that risk of suicide is heightened in early periods of custody, thus strengthening calls for improved screening of suicide risk at reception (Fairweather, 1999; Offender Health Research Network [OHRN], 2008). A recent review showed that the effectiveness of suicide screening tools and checklists is not strong (O'Connor, Gaynes, Burda, Williams, & Whitlock, 2013), with high false-positive and false-negative rates (National Institute for Clinical Excellence, 2011). Nevertheless, their use is generally considered to be an important component of any comprehensive prison suicide prevention policy because it can help identify high-risk groups who might benefit from specific interventions (e.g., treatment for underlying mental health problems) and may reduce suicide risk (Konrad et al., 2007; Mills & Kroner, 2005). For the purposes of the current article, we analyzed all the findings from our two Oxford studies and tested the sensitivity and specificity of different combinations of statistically significant risk factors. We found that useful screening items for male prisoners included questions about: current suicidal ideation, hopelessness, psychiatric disorder, history of psychiatric treatment, previous self-harm or attempted suicide (in prison or outside), family history of suicide and/or self-harm, poor social support, recent homelessness, having been in local authority care before the age of 16 years, and prior incarceration. For female prisoners, the best model included the following factors: remand status (awaiting trial or sentencing), being in custody for a violent offence, current suicidal ideation, hopelessness, psychiatric disorder, previous self-harm or attempted suicide (in prison or outside), history of psychiatric treatment, family history of suicide, poor social support, and having experienced the death of a partner or child. Further research is needed to test the reliability and predictive validity of these instruments.

Repeated risk assessments after the first month following prison arrival should also be considered. Around three quarters of men and women in the Oxford studies of near-fatal self-harm had carried out their attempts over a month after their first reception into custody (Marzano, Hawton, et al., 2011; Rivlin, Hawton, et al., 2013). We would therefore particularly recommend that a reassessment is considered when there are changes in prisoners' circumstances. This may include transfer to a different establishment (Marzano, Hawton, et al., 2011; Rivlin, Hawton, et al., 2013), release from custody (Pratt, Piper, Appleby, Webb, & Shaw, 2006; Zlodre & Fazel, 2012), and other significant life events, which may not necessarily be prison-related (e.g., bereavement, breakdown of relationship; Borrill et al., 2005; Marzano, Fazel, et al., 2011; Rivlin et al., 2011; Suto & Arnaut, 2010). In some countries this is considered to be standard practice, but more research is needed to verify the nature, quality, and effectiveness of such risk reassessments procedures.

### Identification Versus Management of Suicide Risk

Suicide risk appeared to have been correctly identified in almost all women prisoners who made near-lethal suicide attempts in one study (Marzano et al., 2010). While this may relate to more women being repeaters of self-harm (Hawton, Linsell, Adeniji, Sariaslan, & Fazel, 2014), it also demonstrates that – notwithstanding the importance of early and ongoing risk identification – further measures are necessary. Studies of completed suicide in prisons also support this. For example, in a recent study in England and Wales male and female prisoners who had died by suicide between 2005 and 2008 were over nine times more likely to have been identified as being at risk during their prison term than matched controls (Humber et al., 2013).

All the studies reviewed identified multiple risk factors and vulnerabilities of prisoners making near-lethal attempts. This would suggest that no single intervention or approach is likely to be effective on its own. The existing evidence points to the importance of two main areas for intervention: (a) treatment and management of psychiatric disorders and psychosocial problems, and (b) changes to the prison regime and environment. 

### Treatment and Management of Psychiatric Disorders and Psychosocial Problems

Studies have shown discrepancies between the proportions of prisoners with psychiatric problems and those receiving pharmacological and/or psychological interventions (Marzano et al., 2010; Rivlin et al., 2010; see also Fruehwald, Frottier, Matschnig, & Eher, 2003). This calls for continued emphasis on the treatment and management of psychiatric disorders in prisons (Birmingham, 2003; Wilper et al., 2009), especially of depression. The latter appears to be the disorder with the strongest association with both near-lethal attempts and suicide in prisons (Daniel, 2006; Marzano et al., 2010; Rivlin et al., 2010; Suto & Arnaut, 2010). More research is needed to chart the range, use, and effectiveness of prison-based pharmacological, psychosocial, and combined interventions for this and other disorders.

Another area that may warrant particular attention is how best to support prisoners, especially women, who have suffered abuse and bereavement and are experiencing symptoms of PTSD. This may involve therapeutic interventions, including trauma-focused cognitive behavioral therapy for those with severe posttraumatic symptoms (Hudson, 2011; National Collaborating Centre for Mental Health, 2005), and access to other forms of specialist support and information materials (e.g., bereavement guides; Public Health England & the National Suicide Prevention Alliance, 2015). 

The findings of our systematic literature review also suggest that therapeutic interventions aimed at reducing hopelessness and impulsive behaviors should be considered. In the UK, several offending behavior programs (accredited psychosocial interventions, mostly including cognitive-behavioral and problem-solving elements; Hollin, Palmer, & McMurran, 2008) broadly share these aims. However, their impact on prisoners' levels of distress, suicidality and self-harm is seldom assessed (an unpublished exception is Wilson & Borrill, 2005). In UK prisons, psychological interventions specifically targeting these outcomes are relatively rare and poorly evaluated, with the limited interventions available mostly focusing on juveniles and young offenders and on female prisoners (Townsend et al., 2010). Examples include "Carousel," an 8-week group-treatment program for female prisoners (HM Prison Service, 2008); the "Women Offenders Repeated Self-harm Intervention Pilot," a targeted intervention using psychodynamic interpersonal therapy (Shaw & Humber, 2010); pilot programs of dialectical behavioral therapy for female offenders diagnosed with borderline personality disorder (Nee & Farman, 2005); and ACCESS, a group-based intervention aiming to reduce self-harm and bullying among juvenile offenders (Mitchell, Trotter, & Donlon, 2002). Research evaluating these programs and comparable interventions in other countries is limited but ongoing and promising (Eccleston & Sorbello, 2002; Trupin, Stewart, Beach, & Boesky, 2002), not least in demonstrating the viability of delivering cognitively based interventions and dialectical behavioral therapy in secure settings (McCann, Ivanoff, Schmidt, & Beach, 2007). Further feasibility and outcome studies are needed to guide the adaptation of these interventions with different groups of offenders, with adequately powered randomized controlled trials to evaluate their safety and effectiveness in prison settings.

#### Comorbidity of Psychiatric Disorders

Given the importance of comorbidity of psychiatric disorders in prisoners making near-lethal suicide attempts, especially depression or PTSD with substance abuse and antisocial personality disorder, measures to address these are needed. It is known that comorbidity greatly increases risk of suicide in community settings (Foster, Gillespie, & McClelland, 1997). While specific interventions may be especially indicated for particular psychiatric disorders or combination of these, some general principles of management are likely to be beneficial. This may include having specialist psychiatric and dual diagnosis service input into all prisons (Fruehwald et al., 2003) as well as improved access to psychological therapies in prisons and prison-specific mental health and treatment guidelines. In addition, recent research has shown that opiate-substitution therapy for opioid-dependent inmates may significantly contribute to reducing the risk of unnatural death in prisoners (Larney et al., 2014). Above all, effective multi-agency work, "throughcare," and community linkage (during and after imprisonment), supported by good communication and information flow between staff, may reduce the number of suicides in prison and upon release (Daniel, 2006; Freeman & Alamo, 2001; Kovasznay et al., 2004). This may help ensure that the needs and vulnerabilities – psychiatric or otherwise – of individual prisoners and subgroups of prisoners are appropriately identified and managed (see also Rivlin, Ferris, et al., 2013), and may assist in directing (scarce) resources where they are most needed. 

### Environmental Interventions and Changes to the Regime

Seven of the eight studies we reviewed reported associations between high-lethality suicidal behavior and factors relating to the prison environment, especially bullying and social isolation. Together with evidence of clustering of self-harm in prisoners (Hawton et al., 2014), this clearly demonstrates the need also to consider prison-based and more targeted strategies that can address environmental factors associated with suicide risk and increase factors likely to be protective. The latter could include measures aiming to promote purposeful activity (Leese, 2006), meaningful social support and interaction, and the prison's "moral performance" more generally (Liebling & Arnold, 2004), as well as anti-bullying interventions (Ireland, 2002). Specific examples include: the use of shared accommodation (subject to risk assessment); encouraging access to specially trained prisoner "buddies," "insiders," or "listeners" (Hall & Gabor, 2004; Junker, Beeler, & Bates, 2005) and telephone help-lines; facilitating family contact and, where appropriate, their involvement in the risk management/care planning process; creating first-night centers (dedicated units for prisoners who have just arrived into custody) and specialized units for the safe treatment and management of prisoners who are substance dependent.

#### Access to Means of Suicide

Prisoners who attempted suicide by hanging in the Oxford studies, and in studies of completed suicide (Shaw, Baker, Hunt, Moloney, & Appleby, 2004), often used bedding that tears easily to form nooses and was available in a "safer cell" with reduced ligature points (Gunnell, Bennewith, Hawton, Simkin, & Kapur, 2005; in our own research, 23 male prisoners and five females attempted suicide by hanging/ligaturing, despite being in a safer cell). This suggests that a further review of materials used to form nooses is warranted. 

Further ways of reducing access to means of suicide may involve limiting unsupervised access to lethal materials and a risk assessment procedure to assess the safety of allowing a prisoner to keep their own medication. 

#### Training, Support, and Supervision for Prison Officers and Staff

Findings from three of the studies reviewed (Borrill et al., 2005; Marzano, Fazel, et al., 2011; Rivlin et al., 2011) indicate that many prisoners felt that the level of care they received following their self-harm was inadequate, and that being able to speak to someone (including staff) might have helped to prevent their act. Increasing provision for training, support, and supervision for prison officers and other staff (including healthcare practitioners) involved in the care of prisoners at risk may lead to improved staff attitudes and better responses and aftercare following a suicide attempt (Marzano, Ciclitira, & Adler, 2012), and may also help improve their ability to identify those at risk of suicide (Bailey, McHugh, Chisnall, & Forbes, 2000; Hayes, Shaw, Lever-Green, Parker, & Gask, 2008).

### Strengths and Limitations

The recommendations made in this paper are mostly based on findings of eight recent studies on near-lethal suicide attempts in custody. Despite this, and as argued elsewhere, this novel approach offers a number of advantages and has allowed for the identification of the key role of psychiatric comorbidity and psychosocial factors as well as of characteristics traditionally associated with prisoner suicide in psychological autopsy studies (Fazel et al., 2008; Shaw & Turnbull, 2006). In addition, while previous research has mostly lacked power to investigate the contribution of specific diagnostic categories, life events, or psychosocial problems, studies of severe attempts enable more specific and targeted recommendations to be made, particularly in relation to the management of psychiatric disorders and psychosocial problems. Indeed, the studies reviewed demonstrate the value of learning from prisoner near-deaths, as well as completed suicides – not only in a research context, but also potentially as part of formal, and ideally independent, investigations (as is the case in Northern Ireland; The Prisoner Ombudsman for Northern Ireland, 2013). 

One limitation of studies of near-lethal suicide attempts is that they are mostly cross-sectional, interview-based, and reliant on self-report. Some of the information provided by participants may benefit from external corroboration, and further prospective studies are needed to confirm that reported associations with near-lethal self-harm do represent causal risk factors. In addition, data collected from particular countries and types of establishments may not necessarily be generalizable to other settings and populations. 

Nevertheless, these findings lend support to an increasingly convergent body of literature on suicidal behavior in prisoners (see, e.g., Fazel et al., 2008; Sarchiapone, Carli, Di Giannantonio, & Roy, 2009). Although further research is necessary inside custody, the wider evidence from which we derive our recommendations is based on related research from completed suicide in prisons and community settings. Many of our recommendations are consistent with those made in earlier studies and are reflected in existing national standards and guidelines for suicide prevention in custodial settings (Daigle et al., 2007; Konrad et al., 2007). Yet, suicide continues to be a leading cause of death in prisoners. The recent sharp rise in self-inflicted deaths in prisons in England and Wales, following a decline over some years (Humber, Piper, Appleby, & Shaw, 2011; Ministry of Justice, 2015), underlies the importance of suicide prevention policies for prisons, and the need for more research evaluating the feasibility, efficacy, and cost-effectiveness of evidence-based suicide prevention strategies in custodial settings. A recent systematic review of prison-based suicide prevention programs identified only 12 studies evaluating the effectiveness of such interventions, and a great deal of variation in suicide prevention practices around the world (Barker, Kõlves, & De Leo, 2014).

## Conclusion 

Preventing suicide is difficult, especially in a prison setting. While certain aspects of prison life should make suicide more easily preventable than in the community (e.g., by allowing greater monitoring of those at risk, and limiting access to means of suicide), others (e.g., bullying, social isolation, and lack of purposeful activity) may increase risk in an already high-risk population by virtue of their elevated levels of psychiatric morbidity, substance abuse, trauma, and social isolation. The reported impulsivity and high suicidal intent of prisoners' attempts make prison suicides especially difficult to predict. Nevertheless, previous research has shown that comprehensive multifactored suicide prevention programs and – with some caveats – peer-focused suicide prevention initiatives can reduce the number of suicides and suicide attempts in prisons by tackling potentially modifiable environmental, clinical, and psychosocial factors (Barker et al., 2014). 

In this review, we have outlined several interventions that together may improve detection, management, and prevention of suicide in prisoners, and possibly in different subgroups of prisoners. Our findings reiterate calls for a comprehensive but targeted approach, incorporating both population and targeted strategies, individualized care (and throughcare), and multiagency working. Ideally, preventative interventions should address both clinical and prison-related factors, and be sensitive to the needs and vulnerabilities of different groups of prisoners. 

Further research is needed to evaluate and develop key elements of the policies we have put forward, including the proposed risk screening at reception for male and female prisoners. In order to advance theory and practice in this area, it would be helpful if future studies could benefit from the accurate reporting of annual suicide and self-harm rates for all prison services (Fazel et al., 2011), improved links with academic medicine (Kendig, 2004), and more research-friendly prisons. Taking part in research related to personal suicidal behavior does not appear to be distressing for almost all participants even in institutional settings, and can be beneficial in some cases (Rivlin, Hawton, Marzano, & Fazel, 2012). However, significant further progress in reducing suicides in prisons is unlikely without further investment in supportive interventions such as listening services, treatments for PTSD and other common mental disorders, and staff training to support and supervise those caring for people at risk, as well as evaluation of initiatives and other research.

## References

[c1] Bailey J., McHugh M., Chisnall L., & Forbes D. (2000). Training staff in suicide awareness In Towl G., Snow L., & McHugh M. (Eds.), *Suicide in Prison*. Oxford, UK: BPS Blackwell.

[c2] Baillargeon J., Penn J. V., Thomas C. R., Temple J. R., Baillargeon G., & Murray O. J. (2009). Psychiatric disorders and suicide in the nation's largest state prison system. *Journal of the American Academy of Psychiatry and the Law*, , 188–193. 19535556

[c3] Barker E., Kõlves K., & De Leo D. (2014). Management of suicidal and self-harming behaviors in prisons: Systematic literature review of evidence-based activities. *Archives of Suicide Research*, , 178–184. 10.1080/13811118.2013.824830 24611725

[c4] Berman G., & Dar A. (2013). *Prison population statistics*. London, UK: House of Commons Library.

[c5] Birmingham L. (2003). The mental health of prisoners. *Advances in Psychiatric Treatment*, (3), 191–199. 10.1192/apt.9.3.191

[c6] Blaauw E., Kerkhof A., & Winkel F. W. (2001). Identifying suicide risk in penal institutions in the Netherlands. *The British Journal of Forensic Practise*, (4), 22–28.

[c7] Bonner R. L. (2006). Stressful segregation housing and psychosocial vulnerability in prison suicide ideators. *Suicide and Life-Threatening Behavior*, , 250–254. 10.1521/suli.2006.36.2.250 16704328

[c8] Borrill J., Snow L., Medlicott D., Teers R., & Paton J. (2005). Learning from near misses: Interviews with women who survived an incident of severe self-harm. *Howard League Journal*, , 57–69.

[c9] Bradley (2009). *The Bradley Report: Lord Bradley's review of people with mental health problems or learning disabilities in the criminal justice system*. London, UK: Crown. 10.1080/17449200903115847 25757423

[c10] Cinosi E., Martinotti G., De Risio L., & Giannantonio M. Di. (2013). Suicide in prisoners: An italian contribution. *The Open Criminology Journal*, , 18–29.

[c11] Daigle M. S., Daniel A. E., Dear G. E., Frottier P., Hayes L. M., Kerkhof A., … Sarchiapone M. (2007). Preventing suicide in prisons, Part II. *Crisis*, (3), 122–130. 10.1027/0227-5910.28.3.122 17992825

[c12] Daniel A. E. (2006). Preventing suicide in prison: A collaborative responsibility of administrative, custodial, and clinical staff. *Journal of the American Academy of Psychiatry and the Law*, (2), 165–175. 16844795

[c13] Dooley E. (1990). Prison suicide in England and Wales: 1972-1987. *British Journal of Psychiatry*, , 218–221. 10.1192/bjp.156.1.40 2256964

[c14] Eccleston L., & Sorbello L. (2002). The RUSH program – real understanding of self-help: A suicide and self-harm prevention initiative within a prison setting. *Australian Psychologist*, , 237–244. 10.1080/00050060210001706926

[c15] Fairweather C. B. (1999). *Punishment first verdict later: A review of conditions for remand prisoners in Scotland at the end of the 20th century*. Edinburgh, UK: Scottish Executive.

[c16] Fazel S., Cartwright J., Norman-Nott A., & Hawton K. (2008). Suicide in prisoners: A systematic review of risk factors. *Journal of Clinical Psychiatry*, , 1721–1731. 19026254

[c17] Fazel S., Grann M., Kling B., & Hawton K. (2011). Prison suicide in 12 countries: An ecological study of 861 suicides during 2003-2007. *Social Psychiatry and Psychiatric Epidemiology*, (3), 191–5. 10.1007/s00127-010-0184-4 20140663

[c18] Fazel S., Wolf A., & Geddes J. (2013). Suicide in prisoners with bipolar disorder and other psychiatric disorders: A systematic review. *Bipolar Disorders*, (5), 491–495. 23437982 10.1111/bdi.12053

[c19] Foster T., Gillespie K., & McClelland R. (1997). Mental disorders and suicide in Northern Ireland. *British Journal of Psychiatry*, , 447–452. 9307695 10.1192/bjp.170.5.447

[c20] Freeman A., & Alamo C. (2001). Prevention of suicide in a large urban jail. *Psychiatric Annals*, , 447–452.

[c21] Fruehwald S., Frottier P., Matschnig T., & Eher R. (2003). The relevance of suicidal behaviour in jail and prison suicides. *European Psychiatry*, , 161–165. 12814848 10.1016/s0924-9338(03)00064-6

[c22] Gunnell D., Bennewith O., Hawton K., Simkin S., & Kapur N. (2005). The epidemiology and prevention of suicide by hanging: A systematic review. *International Journal of Epidemiology*, , 422–433. 15659471 10.1093/ije/dyh398

[c23] Hall B., & Gabor P. (2004). Peer suicide prevention in a prison. *Crisis*, (1), 19–26. 15384653 10.1027/0227-5910.25.1.19

[c24] Hawton K., Linsell L., Adeniji T., Sariaslan A., & Fazel S. (2014). Self-harm in prisons in England and Wales: An epidemiological study of prevalence, risk factors, clustering, and subsequent suicide. *Lancet*, (9923), 1147–54. 10.1016/S0140-6736(13)62118-2 PMC3978651 24351319

[c25] Hawton K., & Van Heeringen K. (2009). Suicide. *Lancet*, , 1372–1381. 19376453 10.1016/S0140-6736(09)60372-X

[c26] Hayes A. J., Shaw J., Lever-Green G., Parker D., & Gask D. (2008). Improvements to suicide prevention training for prison staff in England and Wales. *Suicide and Life-Threatening Behavior*, , 708–713. 19152301 10.1521/suli.2008.38.6.708

[c27] HM Prison Service (2008). *Prison service order 4800: Women prisoners*. London, UK: Author.

[c28] Hollin C. R., Palmer E. J., & McMurran M. (2008). *Offending behaviour programmes: Development, application, and controversies*. Chichester, UK: John Wiley & Sons 10.1002/9780470713341

[c29] Hudson P. (2011). Effective treatments for PTSD: Practice guidelines from the International Society for Traumatic Stress Studies. *British Journal of Guidance & Counselling*, (2), 194–195.

[c30] Humber N., Piper M., Appleby L., & Shaw J. (2011). Characteristics of and trends in subgroups of prisoner suicides in England and Wales. *Psychological Medicine*, (11), 2275–2285. 21557891 10.1017/S0033291711000705

[c31] Humber N., Webb R., Piper M., Appleby L., & Shaw J. (2013). A national case–control study of risk factors for suicide among prisoners in England and Wales. *Social Psychiatry and Psychiatric Epidemiology*, , 1177–85. 10.1007/s00127-012-0632-4 23232691

[c32] Ireland J. (2002). *Bullying among prisoners: Evidence, research and intervention strategies*. London, UK: Brunner-Routledge.

[c33] Jenkins R., Bhugra D., Meltzer H., Singleton N., Bebbington P., Brugha T., … Paton J. (2005). Psychiatric and social aspects of suicidal behaviour in prisons. *Psychological Medicine*, (2), 257–269. 10.1017/s0033291704002958 15841683

[c34] Junker G., Beeler A., & Bates J. (2005). Using trained inmate observers for suicide watch in a federal correctional setting: A win-win solution. *Psychological Services*, , 20–27.

[c35] Kariminia A., Law M. G., Butler T. G., Corben S. P., Levy M. H., Kaldor J. M., & Grant L. (2007). Factors associated with mortality in a cohort of Australian prisoners. *European Journal of Epidemiology*, , 417–428. 17668280 10.1007/s10654-007-9134-1

[c36] Kendig N. E. (2004). Correctional health care systems and collaboration with academic medicine. *JAMA*, , 501–503. 15280350 10.1001/jama.292.4.501

[c37] Konrad N., Daigle M. S., Daniel A. E., Dear G. E., Frottier P., Hayes L. M., … Sarchiapone M. (2007). Preventing suicide in prisons, part I. Recommendations from the International Association for Suicide Prevention Task Force on Suicide in Prisons. *Crisis*, (3), 113–121. 17992824 10.1027/0227-5910.28.3.113

[c38] Kovasznay B., Miraglia R., Beer R., & Way B. (2004). Reducing suicides in New York State correctional facilities. *Psychiatric Quarterly*, (1), 61–70. 14992303 10.1023/b:psaq.0000007561.83444.a4

[c39] Larney S., Gisev N., Farrell M., Dobbins T., Burns L., Gibson A., & Degenhardt L. (2014). Opioid substitution therapy as a strategy to reduce deaths in prison: Retrospective cohort study. *BMJ Open*, (4), e004666. 10.1136/bmjopen-2013-004666 PMC3987723 24694626

[c40] Leese M. M. (2006). An ecological study of factors associated with rates of self-inflicted death in prisons in England and Wales. *International Journal of Law and Psychiatry*, (5), 355–360. 16780949 10.1016/j.ijlp.2005.10.004

[c41] Liebling A., & Arnold H. (2004). *Prisons and their moral performance: A study of values, quality and prison life*. Oxford, UK: Clarendon Press.

[c42] Lohner J., & Konrad N. (2006). Deliberate self-harm and suicide attempt in custody: Distinguishing features in male inmates' self-injurious behavior. *International Journal of Law and Psychiatry*, (5), 370–385. 16782200 10.1016/j.ijlp.2006.03.004

[c43] Magaletta P. R., Patry M. W., Wheat B., & Bates J. (2008). Prison inmate characteristics and suicide attempt lethality: An exploratory study. *Psychological Services*, (4), 351–361.

[c44] Mann J. J. (2003). Neurobiology of suicidal behaviour. *Nature Reviews Neuroscience*, (10), 819–828. 14523381 10.1038/nrn1220

[c45] Marzano L., Ciclitira K., & Adler J. (2012). The impact of prison staff responses on self-harming behaviours: Prisoners' perspectives. *The British Journal of Clinical Psychology/the British Psychological Society*, (1), 4–18. 10.1111/j.2044-8260.2010.02007.x 22268538

[c46] Marzano L., Fazel S., Rivlin A., & Hawton K. (2010). Psychiatric disorders in women prisoners who have engaged in near-lethal self-harm: Case-control study. *The British Journal of Psychiatry*, (3), 219–26. 10.1192/bjp.bp.109.075424 20807968

[c47] Marzano L., Fazel S., Rivlin A., & Hawton K. (2011). Near-lethal self-harm in women prisoners: Contributing factors and psychological processes. *Journal of Forensic Psychiatry & Psychology*, (6), 863–884. 10.1080/14789949.2011.617465

[c48] Marzano L., Hawton K., Rivlin A., & Fazel S. (2011). Psychosocial influences on prisoner suicide: A case-control study of near-lethal self-harm in women prisoners. *Social Science & Medicine (1982)*, (6), 874–83. 10.1016/j.socscimed.2010.12.028 21345561

[c49] Marzano L., Rivlin A., Fazel S., & Hawton K. (2009). Interviewing survivors of near-lethal self-harm: A novel approach for investigating suicide amongst prisoners. *Journal of forensic and legal medicine*, (3), 152–155. 19239967 10.1016/j.jflm.2008.08.011

[c50] McCann R. A., Ivanoff A., Schmidt H., & Beach B. (2007). Implementing dialectical behavior therapy in residential forensic settings with adults and juveniles In Dimeff L. A. & Koerner K. (Eds.), *Dialectical behavior therapy in clinical practice: Applications across disorders and settings* (pp. 112–144). New York, NY: Guilford Press.

[c51] Mills J. F., & Kroner D. G. (2005). Screening for suicide risk factors in prison inmates: Evaluating the efficiency of the Depression, Hopelessness and Suicide Screening Form (DHS). *Legal and Criminological Psychology*, , 1–12. 10.1348/135532504X15295

[c52] Ministry of Justice (2015). *Safety in custody - quarterly update to September 2014*. London, UK: Author.

[c53] Mitchell J., Trotter G., & Donlon L. (2002). ACCESS-working to reduce self-harm and bullying among juvenile offenders. *Prison Service Journal*, , 31–35.

[c54] Moher D., Liberati A., Tetzlaff J., & Altman D. G., & The PRISMA Group (2009). Preferred Reporting Items for Systematic Reviews and Meta-Analyses: The PRISMA Statement. *PLoS Medicine*, (6), e1000097 10.1371/journal.pmed1000097 19621072 PMC2707599

[c55] National Collaborating Centre for Mental Health (2005). *Clinical guideline 26. Post-traumatic stress disorder: The management of PTSD in adults and children in primary and secondary care*. London, UK: National Institute for Clinical Excellence.

[c56] National Institute for Health and Care Excellence (2011). *Self-harm: Longer-term management*. London, UK: Author. 31886959

[c57] Nee C., & Farman S. (2005). Female prisoners with borderline personality disorder: Some promising treatment developments. *Criminal Behaviour and Mental Health*, (1), 2–16. 10.1002/cbm.33 16470495

[c58] O'Connor E., Gaynes B., Burda B. U., Williams C., & Whitlock E. P. (2013). *Screening for suicide risk in primary care: A systematic evidence review for the U.S. Preventive Services Task Force*. Rockville, MD: Agency for Healthcare Research and Quality. 23678511

[c59] Offender Health Research Network (OHRN) (2008). *An evaluation of the reception screening process used within prisons in England and Wales*. Manchester, UK: Author.

[c60] Opitz-Welke A., Bennefeld-Kersten K., Konrad N., & Welke J. (2013). Prison suicides in Germany from 2000 to 2011. *International Journal of Law and Psychiatry*, , 386–389. 10.1016/j.ijlp.2013.06.018 23850339

[c61] Pratt D., Piper M., Appleby L., Webb R., & Shaw J. (2006). Suicide in recently released prisoners: A population-based cohort study. *Lancet*, , 119–123. 10.1016/S0140-6736(06)69002-8 16829295

[c62] Public Health England & the National Suicide Prevention Alliance (2015). *Help is at hand: Support after someone may have died by suicide*. London, UK: Public Health England.

[c63] Rabe K. (2012). Prison structure, inmate mortality and suicide risk in Europe. *International Journal of Law and Psychiatry*, , 222–230. 10.1016/j.ijlp.2012.02.012 22445577

[c64] Rivlin A., Fazel S., Marzano L., & Hawton K. (2011). The suicidal process in male prisoners making near-lethal suicide attempts. *Psychology, Crime & Law*, (4), 305–327.

[c65] Rivlin A., Fazel S., Marzano L., & Hawton K. (2012). Studying survivors of near-lethal suicide attempts as a proxy for completed suicide in prisons. *Forensic Science International*, (1–3), 19–26. 10.1016/j.forsciint.2012.01.022 22306186

[c66] Rivlin A., Hawton K., Marzano L., & Fazel S. (2010). Psychiatric disorders in male prisoners who made near-lethal suicide attempts: Case-control study. *The British Journal of Psychiatry*, (4), 313–9. 10.1192/bjp.bp.110.077883 20884955

[c67] Rivlin A., Ferris R., Marzano L., Fazel S., & Hawton K. (2013). A typology of male prisoners making near-lethal suicide attempts. *Crisis*, (5), 335–347. 23685335 10.1027/0227-5910/a000205

[c68] Rivlin A., Hawton K., Marzano L., & Fazel S. (2013). Psychosocial characteristics and social networks of suicidal prisoners: Towards a model of suicidal behaviour in detention. *PloS One*, (7), e68944 10.1371/journal.pone.0068944 23922671 PMC3726684

[c69] Rivlin A., Marzano L., Hawton K., & Fazel S. (2012). Impact on prisoners of participating in research interviews related to near-lethal suicide attempts. *Journal of Affective Disorders*, (1–2), 54–62. 10.1016/j.jad.2011.09.009 21975135

[c70] Sarchiapone M., Carli V., Di Giannantonio M., & Roy A. (2009). Risk factors for attempting suicide in prisoners. *Suicide and Life-Threatening Behavior*, (3), 343–350. 19606925 10.1521/suli.2009.39.3.343

[c71] Shaw J., Baker D., Hunt I. A., Moloney A., & Appleby L. (2004). Suicide by prisoners. *British Journal of Psychiatry*, , 263–267. 10.1192/bjp.184.3.263 14990526

[c72] Shaw J., & Humber N. (2010). Suicide and self-injury in offenders In Towl G. & Crighton D. (Eds.), *Forensic psychology*. (pp. 384–397). Chichester, UK: Blackwell.

[c73] Shaw J., & Turnbull P. (2006). Suicide in custody. *Psychiatry*, (8), 286–288.

[c74] Suto I., & Arnaut G. L. Y. (2010). Suicide in prison: A qualitative study. *The Prison Journal*, (3), 288–312.

[c75] The Prisoner Ombudsman for Northern Ireland (2013). *Report by the Prisoner Ombudsman into the circumstances surrounding the near death of Mr C (aged 30) whilst in the custody of Maghaberry Prison on 19 february 2012*. Belfast, UK: Author.

[c76] Townsend E., Walker D.-M. M., Sargeant S., Vostanis P., Hawton K., Stocker O., & Sithole J. (2010). Systematic review and meta-analysis of interventions relevant for young offenders with mood disorders, anxiety disorders, or self-harm. *Journal of Adolescence*, (1), 9–20. 10.1016/j.adolescence.2009.05.015 19560808

[c77] Trupin E. W., Stewart D. G., Beach B., & Boesky L. (2002). Effectiveness of a dialectical behaviour therapy program for incarcerated female juvenile offenders. *Child and Adolescent Mental Health*, , 121–127. 10.1111/1475-3588.00022

[c78] Wilper A. P., Woolhandler S., Boyd J. W., Lasser K. E., McCormick D., Bor D. H., & Himmelstein D. U. (2009). The health and health care of US prisoners: Results of a nationwide survey. *American Journal of Public Health*, , 666–672. 10.2105/AJPH.2008.144279 19150898 PMC2661478

[c79] Wilson M., & Borrill J. (2005). *The impact of enhanced thinking skills on self-harm in custody* (Unpublished report) London, UK: Her Majesty's Stationary Office.

[c80] World Health Organization (2007). *Preventing suicide in jails and prisons*. Geneva, Switzerland: Author.

[c81] Zlodre J., & Fazel S. (2012). All-cause and external mortality in released prisoners: Systematic review and meta-analysis. *American Journal of Public Health*, (12), e65–e75. 10.2105/AJPH.2012.300764 PMC3519300 23078476

